# Carbon fiber doped thermosetting elastomer for flexible sensors: physical properties and microfabrication

**DOI:** 10.1038/s41598-018-30846-3

**Published:** 2018-08-17

**Authors:** Ajit Khosla, Shreyas Shah, MD Nahin Islam Shiblee, Sajjad Husain Mir, Larry Akio Nagahara, Thomas Thundat, Praveen Kumar Shekar, Masaru Kawakami, Hidemitsu Furukawa

**Affiliations:** 1 Department of Mechanical Systems Engineering, Graduate School of Science and Engineering, Yamagata University, Jonan 4-3-16, Yonezawa, Yamagata, 992-8510 Japan; 2 Advanced Materials and BioEngineering Research Centre (AMBER) & Centre for Research on Adaptive Nanostructures and Nanodevices (CRANN), Trinity College Dublin, The University of Dublin, Dublin, 2 Ireland; 3 Department of Chemical and Biomolecular Engineering, Whiting School of Engineering, Johns Hopkins University, 3400 North Charles Street, Baltimore, MD 21218 USA; 4 Chemical and Biological Engineering, University of Buffalo, New York City, NY 14260 USA; 5 Nanomaterials and Sensors Laboratory, Washington State University Vancouver, Vancouver, Washington 98686 USA

## Abstract

We have developed conductive microstructures using micropatternable and conductive hybrid nanocomposite polymer. In this method carbon fibers (CFs) were blended into polydimethylsiloxane (PDMS). Electrical conductivities of different compositions were investigated with various fiber lengths (50–250 μm), and weight percentages (wt%) (10–60 wt%). Sample composites of 2 cm × 1 cm × 500 μm were fabricated for 4-point probe conductivity measurements. The measured percolation thresholds varied with length of the fibers: 50 wt% (307.7 S/m) for 50 µm, 40 wt% (851.1 S/m) for 150 µm, and 30 wt% (769.23 S/m) for 250 μm fibers. The conductive composites showed higher elastic modulus when compared to that of PDMS.

## Introduction

Carbon materials (CMs) possess a variety of favorable properties which can be exploited for the fabrication of potential novel functional components for microelectromechanical systems (MEMS)^1–4^, electronic skins, human-friendly interactive electronics, and wearable health monitors^5–8^. For example, these materials have tendency to form densely packed 3D conductive structures with large surface area. This is highly desirable in devices such as solar cells and miniaturized batteries, where the performance of the device critically depends on large surface area with minimum footprint^9,10^. The range of different spatial formats of CMs can facilitate new MEMS devices with innovative geometries, which are not possible from traditional substrates such as metals, silicon, and glass. Among the CMs, carbon fiber (CF)^11–13^ is a very attractive substrate for electrical signal transduction in miniaturized electrochemical sensors due to the unique advantageous features such as the small dimensions (5–30 μm), low capacitive current, thermal stability, superior mechanical strength, light weight with a specific strength (strength/density) that can be produced from a roll-to-roll process^14^. All these merits are advantageous when CF serves as the substrate in the fabrication of sensors such as strain^15^, pressure^16^, chemical^17,18^, as well as optical and humidity^19^, and MEMS for harvesting energy devices and MEMS scanning micromirror arrays^20^.

In this study, we have used high-performance pitch-based carbon fibers, which have high strength and high modulus of elasticity (940 GPa) and have high thermal conductivity (800 W/m.k)^21–25^. These features can be exploited in the fabrication of flexible and mechanically robust sensor systems. Moreover, one of the biggest advantage of milled pitch-based carbon fiber is its excellent dispensability in polymers^21,25^ which cannot be obtained with graphene or carbon nanotubes. On the other hand, 0D/1D/2D nanomaterials^26–29^ have high tendency of agglomeration in the PDMS. Such a feature makes the composite fail with the incorporation of cracks. Also, 0D/1D/2D nanomaterials needs surface stabilization which impede the conduction of current. Therefore, we have used pitch-based carbon fibers that are inexpensive and robust for scalable flexible device fabrication.

More recently, conductive polymeric composites have been extensively employed in the fabrication of energy storage devices, batteries, 3D printing materials, electromagnetic shielding, flexible displays and smart sensors^30–34^. Compact network of conductive fillers in a polymeric matrix is essential for the development of the composites with high conductivity^35–37^. The characteristics of carbon fibers, such as, high aspect ratio, high conductivity and non-spherical shape are advantageous to achieve percolation path at lower concentration. Most widely used carbon-based polymer composites include polyamides, polymethylmethacrylate (PMMA), acrylonitrile butadiene styrene (ABS), and polyvinylalchol (PVOH). However, many of these are either not flexible and/or difficult to fabricate in micro-scaled features. Therefore, exploration of a flexible materials with better mechanical strength are of great interest.

PDMS is a silicone-based elastomer, which has been widely used as the elastomeric substrate for stretchable electronics. It has been easily and extensively employed for micromolding and fast prototyping of microdevices, such as in the fabrication of microfluidic chip and the stamping for pattern transfer via soft lithography^38–40^. Owing to its diverse properties such as transparency, biocompatibility and flexibility, it has been widely used for fabricating micromixers, microchannels, pumps etc. However due to its non-conductive behavior, the incorporation of electrical functionality has lagged. PDMS is very difficult to pattern conductive pattern lines on it, due to its poor adhesion with metal and conductive polymers^41,42^. Therefore, the integration of the conducting patterns on PDMS is a critical issue, for the fabrication of microsensors, actuators and micropumps, which need electrodes for signal detection. The incorporation of conductive lines is extremely important for signal routing, signal processing electronics and in some cases to supply power. Therefore, in this paper we present a flexible, conductive PDMS composites by incorporating micrometer size pitch-based milled CF. Moreover, we could construct 3D conducting microstructures with excellent mechanical strength incorporated into the PDMS.

## Methods

### Materials

Polydimethylsiloxane (Slygard 184 Silicone Elastomer Kit), consist of Slygard elastomer base and Slygard elastomer curing agent was purchased from Dow Corning corporation, Midland MI, USA. Milled CFs of length 50 µm, 150 µm and 250 µm were purchased from Nippon Graphite Fiber Co. (Tokyo, Japan).

### Fabrication

**Figure 1:**
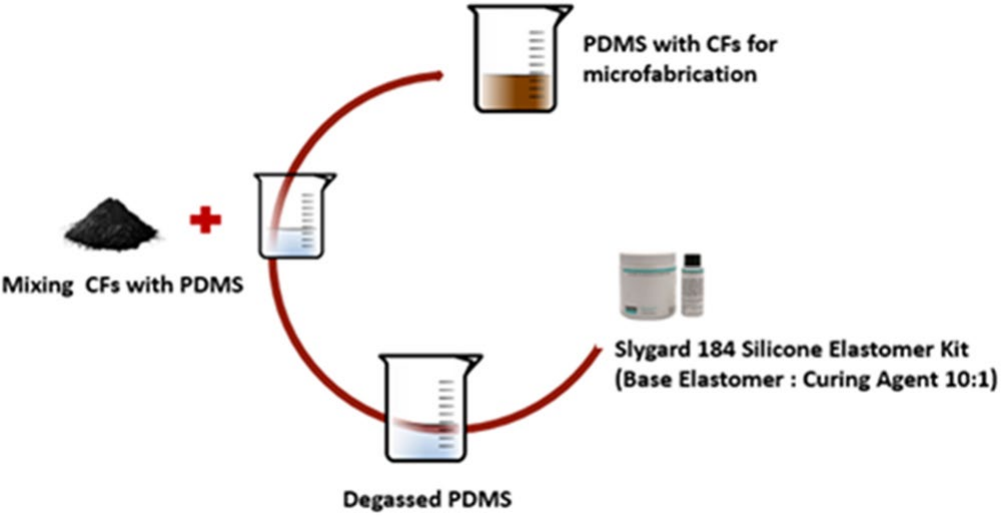
Sample Preparation: Flow shows the process of sample preparation from Silicone elastomer kit mixing base elastomer to curing agent and degassing the bubbles and adding Carbon Fibers to PDMS and mixing it.

Conductive hybrid polymer was prepared using PDMS from elastomer kit by mixing base elastomer and curing agent in ratio of 10:1 weight ratio and degassed to get rid of the bubbles formed during the mixing using a vacuum desiccator for 30 min at room temperature (Fig. 1). Carbon fibers with different wt% (10, 15, 25, 30, 40, 50, and 60) were added and stirred manually in the PDMS matrix. Further, sample were made in the rectangular shape of 2 cm × 1 cm size, and heat pressed at 120 °C at 10 Pa for 3 min to achieve a final thickness of 500 μm.

### Microfabrication of Carbon Fiber PDMS Nano composites

**Figure 2:**
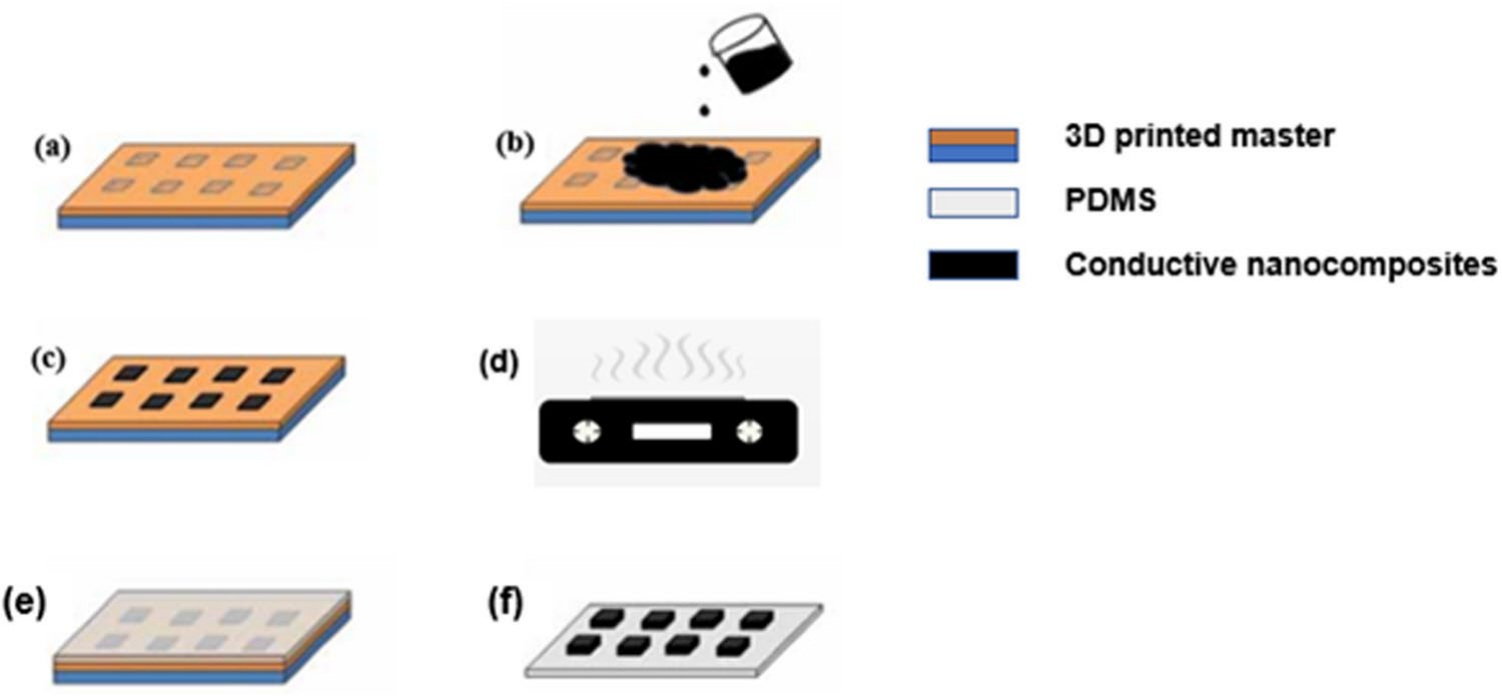
Microfabrication process. (**a**) 3D printed master with micro features b) electrically conductive nanocomposites poured on master (**c**) excess nanocomposites scrapped off the master (**d**) curing the composites on the heating plate (**e**) pouring PDMS on the master (**f**) PDMS-PDMS nanocomposites peeled off the master.

PDMS-based electrically conductive composite were micropatterened using hybrid fabrication process shown in Fig. 2. A 3D- printed master with negative tone features, was fabricated using an Objet30 Pro 3D printer.

## Results and Discussion

**Figure 3:**
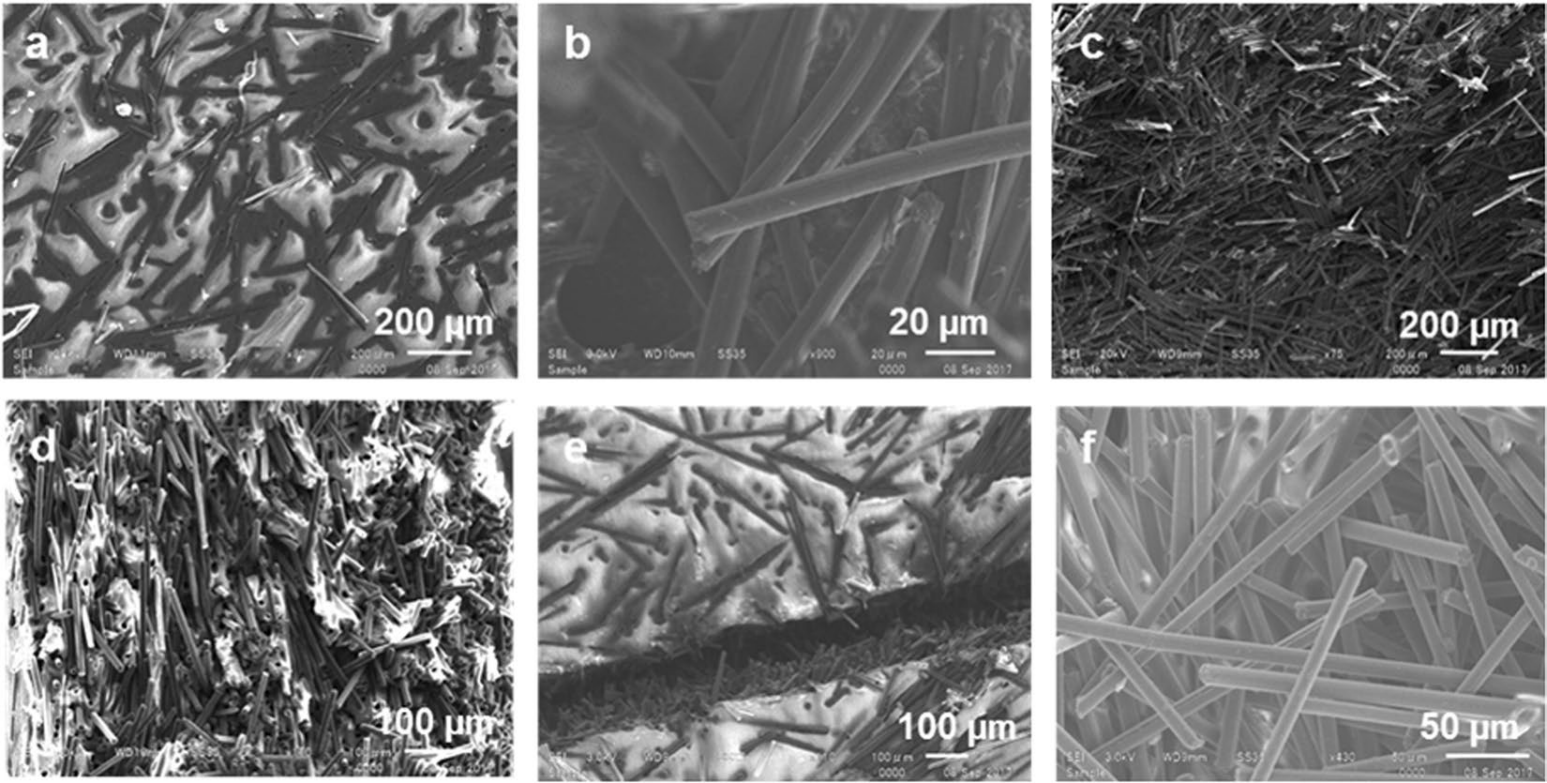
SEM images of the carbon fibers in the PDMS matrix. (**a**) 10 wt% of carbon fiber length, 250 μm sample, (**b**) 15 wt% of carbon fibers, length 150 μm, (**c**) Cross section of 40 wt% of carbon fibers, (**d**) Carbon fibers dispersion along the width of the sample, (**e**) Cut on the surface shows the fibers dispersed in height, (**f**) 50 wt% length and 150 μm sample.

CF with different wt% (10, 15, 40 and 50) were added and stirred manually in the PDMS matrix. Samples were made in the rectangular shape of 2 cm × 1 cm size, and heat pressed at 120 °C at 10 Pa for 3 min. Figure 3 shows scanning electron microscopic (SEM) images of the carbon fibers in the PDMS matrix with different wt% of carbon fiber (average length of 50–250 μm).

**Figure 4:**
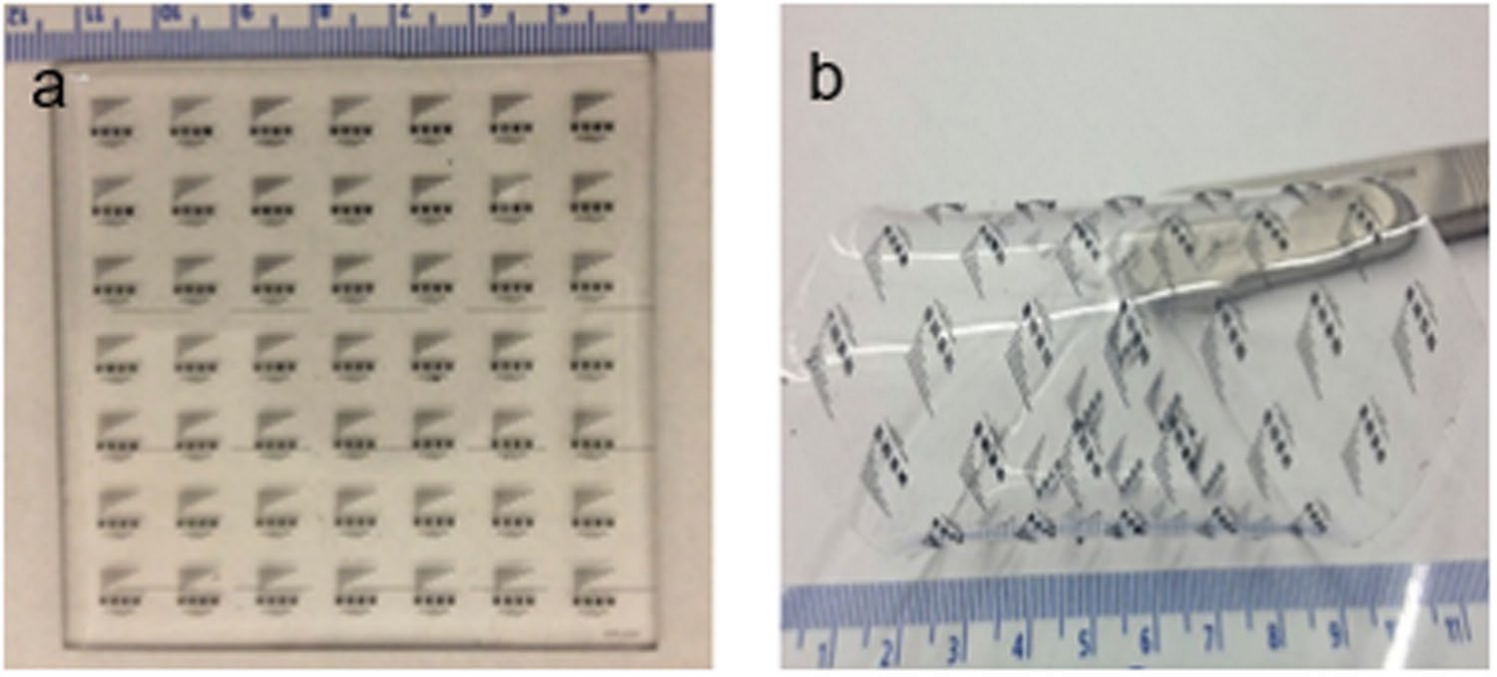
Optical micrographs of the fabricated of conductive features on nonconductive PDMS polymer. (**a**) CFs-PDMS nanocomposite array of micro features (**b**) Flexible conductive features on non-conductive polymer.

Hybrid fabrication process involves pouring electrically conductive nanocomposites on the 3D printed mold and using spin coater at 1000 rpm for 4 min settling the composites on the mold evenly. The excess amount of the composite was wiped off the master using IPA wipes. Undoped PDMS was poured on the master to transfer the conductive features. Hybrid composite was cured at 110 °C under heat plate for 30 min and peel off from the master. This gives the electrically conductive features on the nonconductive PDMS as shown in the Fig. 4.

### Conductivity Measurements

Sample size of 500 µm thickness was fabricated and tested with the 4-point terminal sensing to eliminate contact resistance. 3D printed jig was used to place the sample to measure resistance. Known current was passed through the sample using the input leads, and sense leads measured the voltage through the sample^43^. Resistance between the input and sense leads was calculated using the Ohm’s law. Since no current is withdrawn from the sense leads, contact resistance could be eliminated thus giving bulk resistance. The distance between the leads and sample geometry was known resistivity of the sample and was calculated using Equation 1.

1 $$\rho =Rs\ast t$$

**Figure 5:**
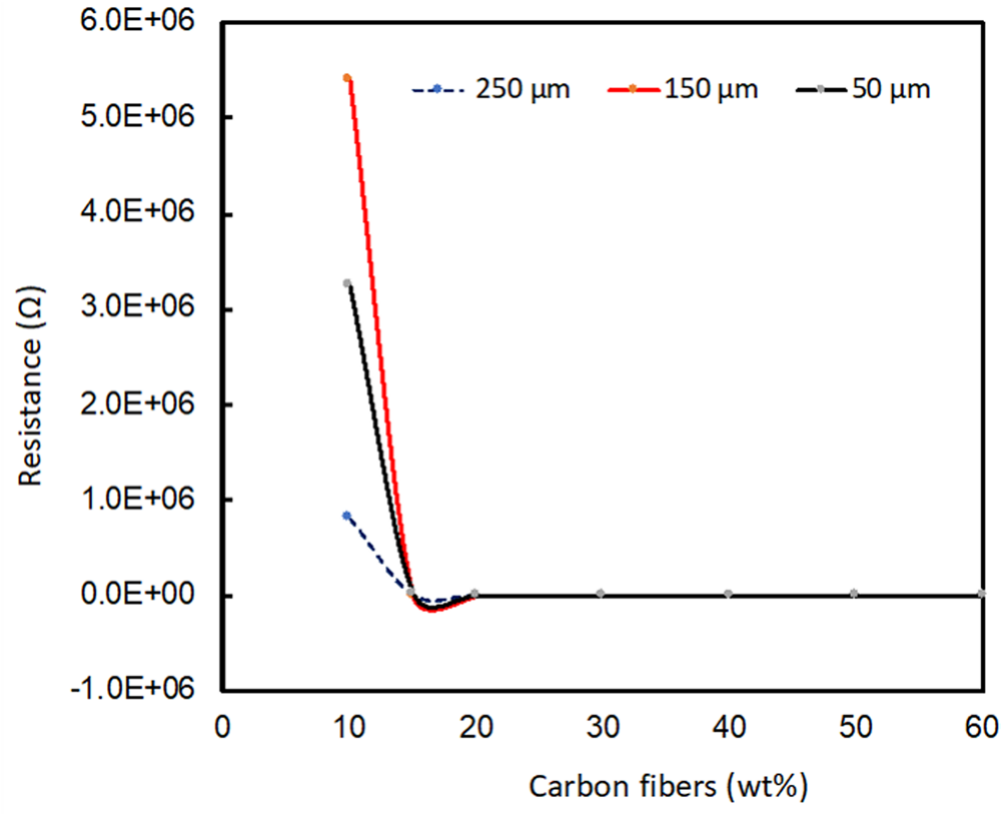
Graphical representation of dependence of resistance on carbon fiber (wt%) for 250, 150 and 50 μm length carbon fibers.

Resistivity (ρ) was calculated by bulk resistance multiplies thickness (t) for variant weight percentage of filler. Samples with different length of the fibers were fabricated and resistance was test was performed as shown in the Fig. 5. From Fig. 5 it can be seen that the resistance values decrease with the increase in the wt% of carbon fibers. Further, Table S1 shows the different values of resistivity as a function of the length of the fibers with the wt% remaining constant.

**Figure 6:**
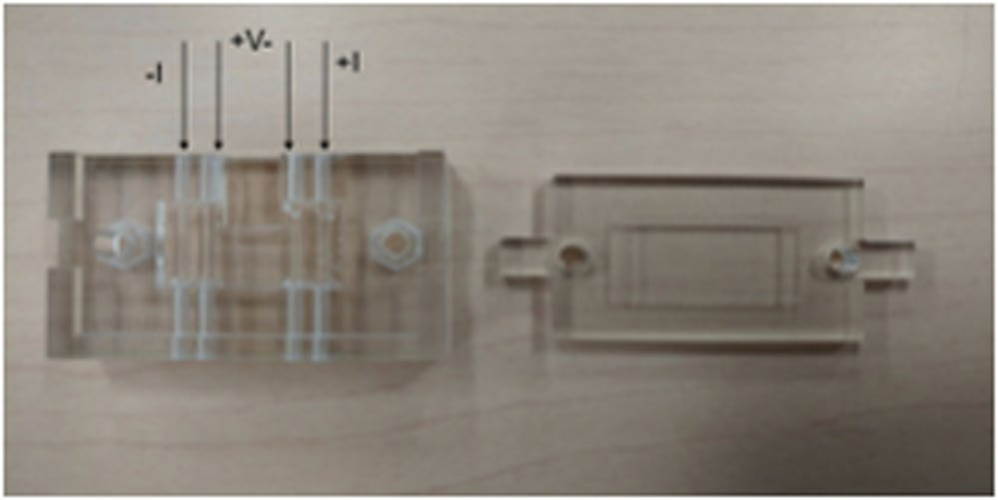
3D printed jig for 4-point sensing measurement.

**Figure 7:**
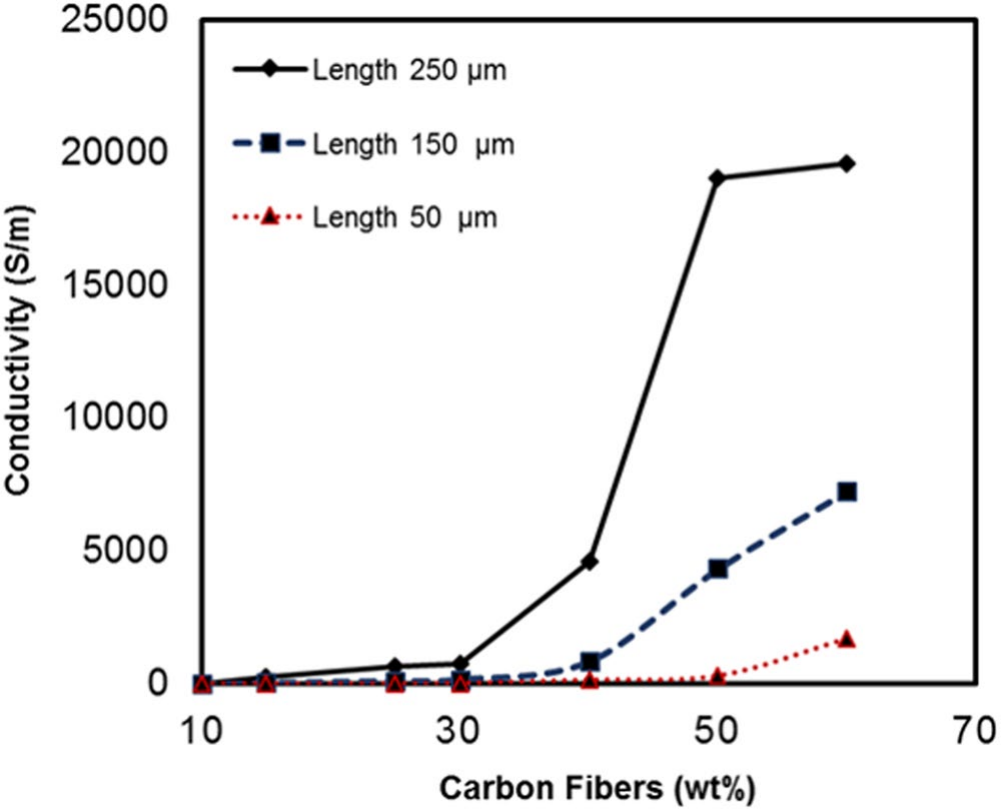
Conductivity versus carbon fibers weight percentage.

The electrical conductivity of CFs in PDMS matrix as a function of wt% was measured using the setup shown in Fig. 6. From Fig. 7, it is observed that the conductivity increases slowly up to 30 wt% for 250 μm length fiber, 40 wt% for 150 μm and 50 wt% for 50 μm. However, conductivity increases swiftly after these points. This variation can be explained in terms of percolation theory. The percolation path is not set up at lower concentration of carbon fibers, as the concentration of fibers increases conducting percolation paths are setup^44,45^. Insulator to conductor transition point on fibers weight percentage plot is referred as percolation threshold. The percolation limit for carbon fiber-PDMS composites for 250 μm, 150 μm and 50 μm length fibers were at 30 wt%, 40 wt% and 50 wt%.

### Mechanical Characterization

#### Thermal gravimetric analysis (TGA)

TGA results for PDMS and hybrid polymer shows the mass reduction as the function of temperature under a nitrogen purge. Approximately, 10 mg of sample was heated at a rate of 10 °C/min. TGA results were calculated three times for each sample which shows that PDMS goes into thermal degradation beginning around 488.53 °C with total mass loss of 74.14%. TGA results of PDMS and carbon fibers shows that due to the incorporation of fibers in the PDMS matrix enhance the thermal stability of composite. However, thermal degradation of PDMS and PDMS-Carbon fibers 15 wt% were left with around 25% of the total weight. Whereas 77–80% weight was remaining for PDMS-Carbon fibers 30 wt% and 50 wt% at around 780 °C as shown in Fig. S1. Furthermore, in PDMS-Carbon fibers composites starts degrading at <170 °C and weight loss of 5–7% occurred at 250 °C. The first derivative of thermo-gravimetric (DTG) shows the small shoulder on the TGA. DTG plot confirms the thermal degradation at 480 °C and 780 °C for the composites^46^.

#### Differential Thermal Analysis (DTA)

**Figure 8:**
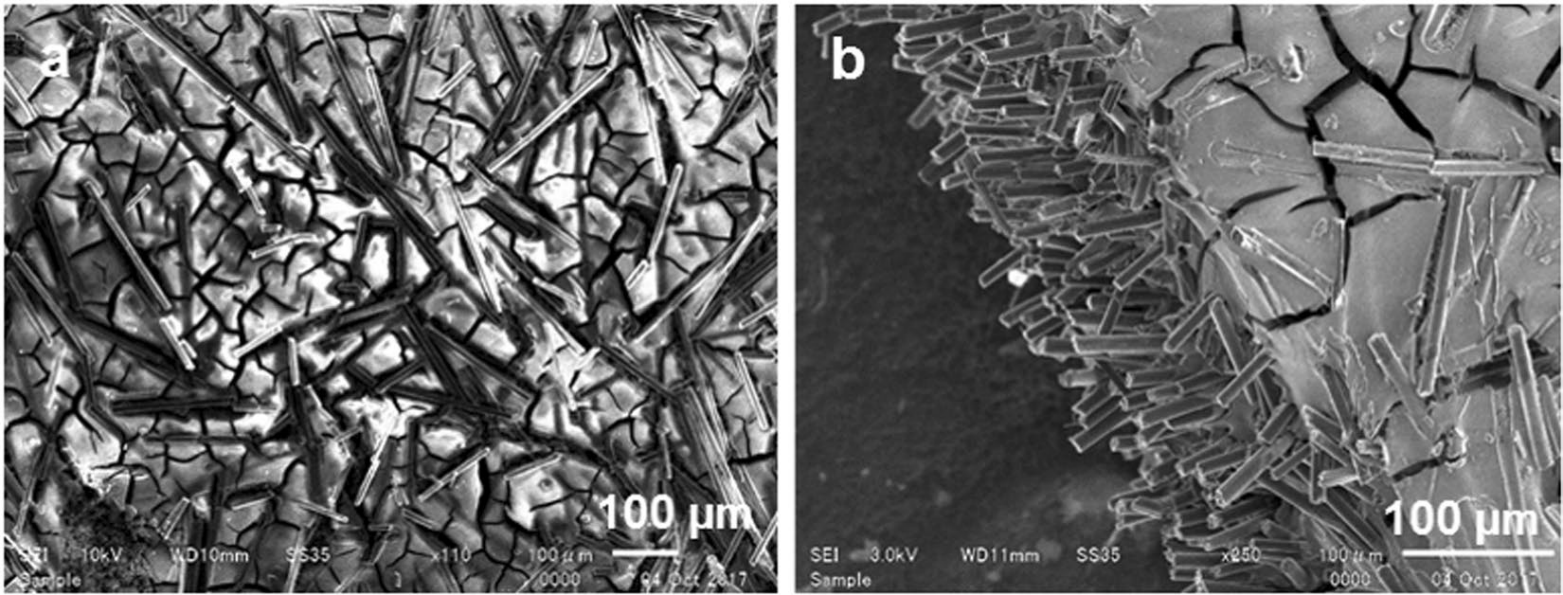
SEM images of samples post TGA (**a**) 250 μm length fiber dispersed in 15 wt%, (**b**) 150 μm length samples dispersed with 50 wt% in PDMS

Further, (DTA-Heat flow) results shows the PDMS and PDMS-carbon fiber samples exhibits small endothermic peak at 180 °C showing loss of adsorbed water as shown in Fig. S2. After a gradual weight loss, a sharp weight loss was observed accompanied by the large broad exothermic peak shows in the DTA curve suggesting the decomposition of some organic compounds. After ∼620 °C the weight loss remained approximately half of the initial weight and the DTA analysis proved endothermic reaction. This could be ascribed to the PDMS weight rapidly decreased after 400 °C which accompanied by large shrinkage and even cracks in the composite (Fig. 8). The overall weight loss is higher in the samples with lower CFs content.

#### Dynamic Mechanical Analysis (DMA)

**Figure 9:**
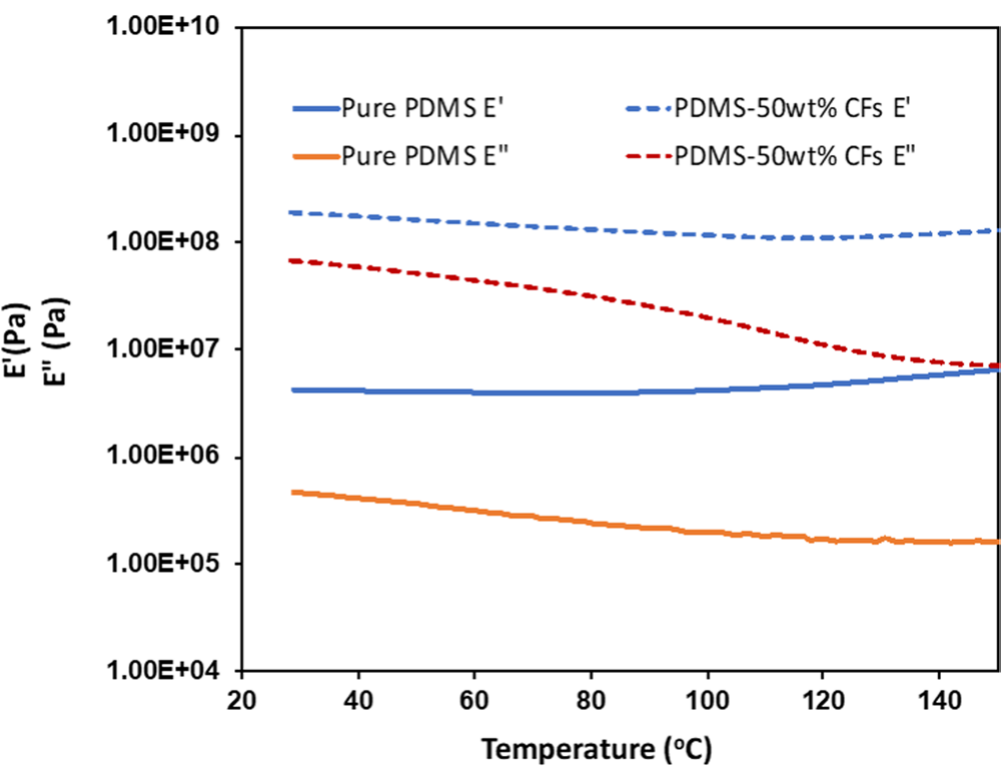
DMA plot for pure PDMS and nanocomposites with 50 wt% CF loading showing storage modulus and loss modulus Vs temperature at 10 Hz.

To investigate the mechanical properties of the hybrid polymer at varying temperature conditions, DMA was carried out from room temperature to 150 °C. DMA results shown in Fig. 9 for pure PDMS and PDMS-carbon fibers composites demonstrates storage modulus (E′) and loss modulus (E′′) against temperature at frequency of 10 Hz. The storage modulus of pure PDMS has been found to be about 45 times lower than that of PDMS 50 wt% CFs composites. Storage modulus for both cases has been found to remain almost constant throughout the whole temperature region suggesting good mechanical performance at elevated temperature on the other hand loss moduli for both cases showed monotonous decrease with increase in temperature signifying decreased elasticity at elevated temperature. For both samples loss moduli are lower than the storage moduli where loss modulus of PDMS 50 wt% CF has been found higher than the pure PDMS. So, it can be said that doping CFs in PDMS matrix the mechanical properties can be improved to a large extent.

## Conclusion

In conclusion, we demonstrated the microfabrication of electrically conductive polymer. Further, we investigated the electrical and mechanical properties as well as the carbon fibers dispersion of carbon fibers/PDMS composites for varying carbon fiber weight ratios. SEM analysis shows that the carbon fibers were dispersed with little agglomeration. It was observed that the percolation threshold for carbon fibers with length 250 μm, 150 μm and 50 μm were observed at 30, 40 and 50 wt% respectively. We compared the thermal stability of PDMS and PDMS-carbon fibers with various wt%. TGA results showed the thermal stability improved with the increase in wt% of carbon fibers and DTA analysis showed the better thermal degradation properties of PDMS-carbon fibers. Hybrid PDMS nanocomposites showed enhancement in elastic modulus than that of pure PDMS. Thus, the developed micropatternable composites can be used to develop flexible and stretchable micro-sensors (chemical and physical), conductive lines or traces, elastomeric printed circuits and flexible sensors for Internet of Things (IoT) and for lab-on-chip devices.

## Electronic supplementary material

Supporting Information
