# Supplementary material for: Carbon fiber doped thermosetting elastomer for flexible sensors: physical properties and microfabrication
Source: Sci Rep. 2018 Aug 17;8:12313. doi: 10.1038/s41598-018-30846-3 (PMC6097983; doi:10.1038/s41598-018-30846-3)
Supplement: Supplementary file 1 — Supporting Information [file 41598_2018_30846_MOESM1_ESM.docx]

Supporting Information

**Carbon fiber doped thermosetting elastomer for flexible sensors: physical properties and microfabrication**

Ajit Khosla^1*^, Shreyas Shah^1^, MD Nahin Islam Shiblee^1^, Sajjad Husain Mir^2^, Larry Akio Nagahara^3^, Thomas Thundat^4^, Praveen Kumar Shekar^5^, Masaru Kawakami^1^, and Hidemitsu Furukawa^1^

^1^ Department of Mechanical Systems Engineering, Graduate School of Science and Engineering, Yamagata University, Jonan 4-3-16, Yonezawa, Yamagata 992-8510, Japan

^2^ Advanced Materials and BioEngineering Research Centre (AMBER) & Centre for Research on Adaptive Nanostructures and Nanodevices (CRANN), Trinity College Dublin, The University of Dublin, Dublin 2, Ireland

^3^ Department of Chemical and Biomolecular Engineering, Whiting School of Engineering, Johns Hopkins University, 3400 North Charles Street, Baltimore, MD 21218, USA

^4^ Chemical and Biological Engineering, University of Buffalo, NY 14260, USA

^5^ Nanomaterials and Sensors Laboratory, Washington State University Vancouver, Vancouver, WA 98686, USA

***** Corresponding author: Ajit Khosla: [khosla@gmail.com](mailto:khosla@gmail.com)

**Table S1**. Bulk resistance and resistivity values of variant wt% of carbon fibers.

| Wt% | 10 | 15 | 20 | 30 | 40 | 50 | 60 | |
| --- | --- | --- | --- | --- | --- | --- | --- | --- |
| Bulk Resistance  4-Point 250 μm Ω | 810000 | 7.5 | 3 | 2.6 | 0.435 | .105 | .102 | |
| Bulk Resistance  4-Point 150 μm Ω | 5400000 | 34.2 | 15.95 | 10.9 | 2.35 | 0.46 | | 0.275 |
| Bulk Resistance  4-Point 50 μm Ω | 3250000 | 27000 | 9850 | 76 | 13.235 | 6.5 | | 1.15 |
| Resistivity 250 μm (Ω m) | 405 | 0.00375 | 0.0015 | 0.0013 | 0.0002175 | 0.0000525 | | 0.000051 |
| Resistivity 150 μm (Ω m) | 2700 | 0.0171 | 0.007975 | 0.00545 | 0.001175 | 0.00023 | | 0.0001375 |
| Resistivity 50 μm  (Ω m) | 1625 | 13.5 | 4.925 | 0.038 | 0.0066175 | 0.00325 | | 0.000575 |


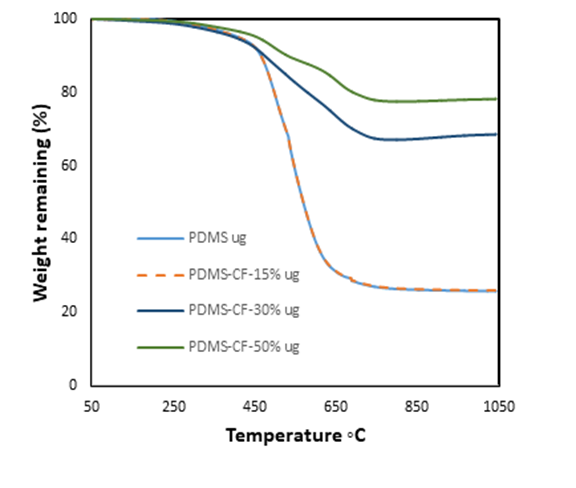


**Figure S1**. TGA anaylsis of PDMS and PDMS-Carbon fibers.


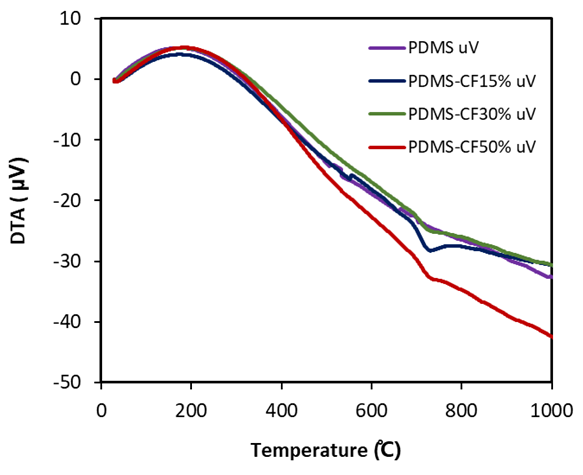


**Figure S2**. DTA anaylsis of hybrid polymer of variant carbon fiber weightage.


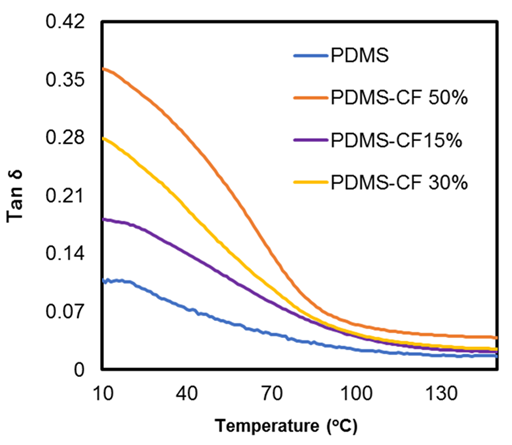


**Figure S3**. Comparison of loss factor with different weight percentage of CFs dispersed in PDMS matrix.

**Table S2**: Comparative studies of electrical/mechanical properties of PDMS-CF and PDMS-milled pitched CF

|  | PDMS-CF (references) | Electrical Resistance (ohm m^-1^) | Tensile strength (Pa) |
| --- | --- | --- | --- |
| 1 | *Composites Part B*, 2016, B94, 102-108 | 0.55 | 26x10^5^ |
| 2 | *ACS* *Appl*. *Mater*. *Interfaces* 2017,9, 14207-4215 | 30 | 314x10^3^ |
| 3 | *RSC Adv*., 2017, 7, 14761-14768 | 5x10^-2^ | Not observed |
| 4 | *Int*. *J*. *Smart NANO Mater*., 2016, 4, 236-247 | 0.012 | Not observed |
| 5 | PLoS ONE 13(2): e0189415 | 100 | 4x10^5^ |
| **6** | **PDMS milled Pitch CF** | **0.00102** | **1x10^8^** |
